# Intracranial glioma xenograft model rapidly reestablishes blood–brain barrier integrity for longitudinal imaging of tumor progression using fluorescence molecular tomography and contrast agents

**DOI:** 10.1117/1.JBO.25.2.026004

**Published:** 2020-02-28

**Authors:** LeMoyne Habimana-Griffin, Dezhuang Ye, Julia Carpenter, Julie Prior, Gail Sudlow, Lynne Marsala, Matthew Mixdorf, Joshua B. Rubin, Hong Chen, Samuel Achilefu

**Affiliations:** a Washington University School of Medicine, Department of Radiology, St. Louis, Missouri, United States; b Washington University, Department of Biomedical Engineering, St. Louis, Missouri, United States; c Washington University, Department of Mechanical Engineering and Materials Science, St. Louis, Missouri, United States; d Washington University School of Medicine, Department of Pediatrics, St. Louis, Missouri, United States; e Washington University School of Medicine, Department of Radiation Oncology, St. Louis, Missouri, United States; f Washington University School of Medicine, Department of Biochemistry and Molecular Biophysics, St. Louis, Missouri, United States

**Keywords:** fluorescence molecular tomography, longitudinal imaging, molecular imaging, glioblastoma, focused ultrasound, indocyanine green, near-infrared fluorescence imaging

## Abstract

**Significance:** The blood–brain barrier (BBB) is a major obstacle to detecting and treating brain tumors. Overcoming this challenge will facilitate the early and accurate detection of brain lesions and guide surgical resection of tumors.

**Aim:** We generated an orthotopic brain tumor model that simulates the pathophysiology of gliomas at early stages; determine the BBB integrity and breakdown over the time course of tumor progression using generic and cancer-targeted near-infrared (NIR) fluorescent molecular probes.

**Approach:** We developed an intracranial tumor xenograft model that rapidly reestablished BBB integrity and monitored tumor progression by bioluminescence imaging. Sham control mice were injected with phosphate-buffered saline only. Fluorescence molecular tomography (FMT) was used to quantify the uptake of tumor-targeted and passive NIR fluorescent imaging agents in orthotopic glioma (U87-GL-GFP PDE7B H217Q cells) tumor model. Cancer-induced and transient (with focused ultrasound, FUS) disruption of BBB integrity was monitored with NIR fluorescent dyes.

**Results:** Stereotactic injection of 50,000 cells into mouse brain allowed rapid reestablishment of BBB integrity within a week, as determined by the inability of both tumor-targeted and generic NIR imaging agents to extravasate into the brain. Tumor-induced BBB disruption was observed 7 weeks after tumor implantation. FUS achieved a similar effect at any time point after reestablishing BBB integrity. While tumor uptake and retention of the passive NIR dye, indocyanine green, was negligible, both actively tumor-targeting agents exhibited selective accumulation in the tumor region. The tumor-targeting molecular probe that clears rapidly from nontumor brain tissue exhibits higher contrast than the analogous vascular-targeting agent and helps delineate tumors from sham control.

**Conclusions:** We highlight the utility of FMT imaging for longitudinal assessment of brain tumors and the interplay between the stages of BBB disruption and molecular probe retention in tumors, with potential application to other neurological diseases.

## 1 Introduction

Glioblastoma (GBM) remains one of the most aggressive and deadly types of cancer with an estimated 5-year survival rate of 5.5%.^1^ It is also the most common type of primary malignant brain tumor with 12,760 cases projected in the United States in 2018.^1^ Management of GBM presents many unique obstacles owing to the relative importance of CNS tissue, necessitating the sparing of tissue during surgical resection. Furthermore, protection of the tumor by the blood–brain barrier (BBB) as well as tumor invasiveness, heterogeneity, and drug resistance limits the effectiveness of systemic therapies.^2^^–^^4^ In the face of these complex challenges, the role of molecular classification of GBM is increasingly important for diagnosing specific disease phenotypes, choosing the best treatment options, and monitoring treatment response.^3^^,^^5^

Molecular imaging can provide insight into basic mechanisms of GBM pathogenesis and evolution as well as assay delivery of diagnostic probes and therapeutic agents. Fundamentally, molecular imaging enables the detection of specific molecules or molecular processes and can be designed to respond to a particular biological target, enzymatic activity, or local environmental factors, such as pH or temperature. Optical imaging techniques, such as fluorescence molecular tomography (FMT), are ideal for preclinical molecular imaging and modeling of disease progression. In addition to offering a wide variety of commercially available molecular probes, high detection sensitivity, and minimal exposure to ionizing radiation, optical methods are generally low cost, portable, and scalable from the microscopic to macroscopic scale compared with traditional imaging systems.^6^ Near-infrared (NIR) FMT is akin to x-ray computed tomography in that it produces three-dimensional (3-D) reconstructions of fluorescence distribution by acquiring multiple fluorescence projection images. This technique is advantageous over other optical imaging techniques that have been previously utilized for molecular imaging of brain tumors, such as fluorescence reflectance imaging and bioluminescence imaging (BLI) because of the acquisition of paired excitation and fluorescence images that account for tissue inhomogeneity and the ability to localize fluorescence in 3-D with absolute quantification.^7^^–^^9^

A combination of high sensitivity, low cost, quantitative, and noninvasive nature of FMT makes it an attractive approach for longitudinal molecular imaging of GBM to understand disease evolution and to monitor treatment response. A recent study tracked longitudinal growth of orthotopic GBM with an infrared fluorescent protein (iRFP),^10^ but this study did not examine how the dynamics of exogenous delivery of contrast agents changes over time when measured by FMT. In this study, we sought to evaluate how the tumor uptake of a passive NIR fluorescent imaging agent, indocyanine green (ICG), and an active tumor-targeting agent, LS301, evolves as tumors progress. ICG has been used in clinical studies for fluorescence-guided resection of GBM ^11^^,^^12^ and is thought to accumulate in tumors through passive delivery to tissues with preferential tumor uptake due to the increased endocytic activity of tumors.^13^ Conversely, LS301 is a cancer-accumulating agent that has been utilized for fluorescence imaging of a variety of tumor types^14^^–^^16^ and targets phosphorylated annexin A2 (pANXA2),^17^ which is overexpressed in many cancers.^18^ Together, ICG and LS301 allow for a reasonable comparison between passive and active targeted probes, given their spectral similarity^19^ and that ICG and the dye component of LS301 (cypate) bind similarly to bovine serum albumin.^20^ In addition, we examine the utility of focused ultrasound (FUS)-enhanced delivery of passive and targeted probes as a means to bypass the BBB for molecular imaging of GBM. Lastly, we compare the tumor-targeting capability of LS301 with transferrin, an established GBM-targeting molecule^21^ to examine how targeting different receptors mediate tumor uptake. Our results demonstrate that accessibility to the tumor is only one step of the process. Retention of molecular probes in tumors, however, is governed by other biological processes that require a selective active internalization of the probes in tumors.

## 2 Materials and Methods

### 2.1 Cell Line and Animal Model

All studies were conducted in compliance with Washington University Animal Welfare Committee’s requirements for the care and use of laboratory animals in research. U87-GL-GFP PDE7B H217Q cells^22^ were cultured in Dulbecco’s Modified Eagle Medium supplemented with 10% FBS, penicillin (100  units/mL), and streptomycin (100  μg/mL), all obtained from Gibco (Life Technologies, New York). Tumors were initiated by stereotactic injection of $5\times 10^{4}\text{ cells}$ in 2-μL phosphate buffered saline (PBS) in the brain (relative to bregma 0.5 mm anterior, 2.2 lateral, 0.6 to 0.65 mm ventral) into Athymic NCr-nu/nu female mice aged 7 to 13 weeks (Charles River Laboratories). Sham control mice were injected with 2  μL of PBS. Mice were maintained on a low-fluorescence chow diet.

### 2.2 Bioluminescence Imaging

For BLI of living animals, mice were injected intraperitoneally with 150  μg/g d-luciferin (Gold Biotechnology, Missouri) in PBS, anesthetized with 2.5% isoflurane, and imaged with a charge-coupled device camera-basedBLI system (IVIS 50 Perkin Elmer, Massachusetts); exposure time 1 to 60 s, binning 4 to 8, field of view 12, f/stop 1, and open filter. Radiance is displayed as $\text{photons}/s/{\mathrm{cm}}^{2}/\mathrm{sr}$.

### 2.3 Fluorescence Molecular Tomography

Mice were anesthetized with 2% isoflurane and injected intravenously with either 60  μM ICG (Cardiogreen, Sigma-Aldrich, Missouri) or Cypate-cyclo(D-Cys-Gly-Arg–Asp-Ser-Pro-Cys)-Lys-OH (LS301) in 100-μL PBS or Alexa Fluor™ 680 Conjugate (ThermoFisher, Massachusetts). 3-D tumor images were obtained using the FMT 4000 system (PerkinElmer, Inc., Massachusetts) with 1.5  mm×1.5  mm source density. Reconstruction and image analyses were performed using TrueQuant™ software (PerkinElmer, Inc., Massachusetts). Fluorescence quantification of fluorophores was based on concentration-dependent calibration using the calibration phantom provided with the system. Rectangular prism ROIs were drawn manually around tumors of 3-D reconstructed images, using the topographic projections, stereotactic coordinates, and bioluminescence images for guidance. Quantification of data is given as mean fluorophore concentration in regions of interest, omitting voxels with arbitrary low signal ($<10^{-9}\;\mathrm{nM}$). Background subtracted quantification was obtained by subtracting the mean concentration in the preinjection data from postinjection data.

### 2.4 Longitudinal Imaging

The U87GL cells (n=8) or PBS for sham controls (n=5) were initiated by stereotactic cortical injection.BLI was used to track tumor progression. Mice were then imaged weekly by BLI and FMT with successive injections of ICG and LS301. Typically, images were acquired preinjection and at 0.5, 1, 4, and 24 h, following each injection using the 790-nm channel of the FMT for up to 7 weeks postcortical injection.

### 2.5 Focused Ultrasound

Sonication was performed as described previously.^23^^,^^24^ A preclinical FUS system (VIFU 2000, Alpinion US Inc., Washington) was used for FUS sonication. The FUS transducer has a center frequency of 1.5 MHz, focal depth of 60 mm, and aperture of 60 mm. The transducer was attached to a water balloon, which was filled with degassed water to provide acoustic coupling. The water balloon was immersed in a degassed-water container. The bottom of the water container had a window sealed with an almost acoustically and optically transparent membrane. The container was placed on the mouse head and coupled with degassed ultrasound gel. The FUS transducer has a circular central opening of 38 mm in diameter. A B-mode imaging probe (L8-17, Alpinion, Seoul, South Korea) was inserted into the opening and aligned with the FUS focal plane. The pressure amplitude of the FUS transducer was calibrated using a needle hydrophone (Onda, California) in a degassed water tank before the *in vivo* experiment. The reported pressure amplitudes were the measured peak negative pressure calculated using the hydrophone measured pressure values attenuating by 18% to correct for mouse skull attenuation. The lateral and axial full-width-at-half maximum pressures of the beam were 6.04 and 0.62 mm, respectively. The FUS transducer was attached to a 3-D positioning system (Velmex, Lachine, Quebec, Canada). In this study, the tumor was targeted with the assistance of a grid. The grid was positioned in the water container on top of the skull with the crossing point in alignment with the tumor in reference to the BLI image and the stereotaxic coordinates of the tumor injection. The B-mode imaging probe was used to scan through the grid and form an image of the grid. Then, the crossing point of the grid was then identified. The depth of FUS was adjusted to be 0.65 mm to the skull by measuring the distance using the B-mode imaging. Five adjacent points (four located at the corner of a square with one in the middle) were targeted to cover the whole tumor. Freshly diluted microbubble suspension (30  μL) was administered through a bolus injection via the tail vein before FUS sonication. Immediately after injection (5 s), pulsed FUS (center frequency: 1.5 MHz; ultrasound pressure: 0.85 MPa; pulse length: 6.7 ms; pulse repetition frequency: 5 Hz; duration: 1 min each point) was applied. LS301 or ICG was injected after each treatment.

### 2.6 Histology

Mouse brains were harvested and frozen in optimal cutting temperature embedding medium (Fisher Healthcare, Texas) and stored at −20°C. Fluorescence and brightfield images of 10  μm sections were obtained by epifluorescence microscopy at 4× and 20× magnifications. Fluorescence images from LS301 were acquired before fixation and immunohistochemistry. Slides were stained with a monoclonal antibody to murine p-ANXA2 (Santa Cruz Biotechnology, Inc., Texas) at a 1:250 dilution, followed by secondary staining with a donkey anti-mouse Alexa Fluor 594 conjugate (ThermoFisher, Massachusetts) at a 1:1000 dilution.

### 2.7 Data Analysis and Statistics

Statistical significance was measured by a two-tailed Student’s t-test using GraphPad Prism software (GraphPad, California). Comparison of longitudinal data included the Holm–Sidak correction for multiple comparisons. All values are means and error bars are standard deviations. *P<0.05, **P<0.01, ***P<0.001.

## 3 Results

**Fig. 1:**
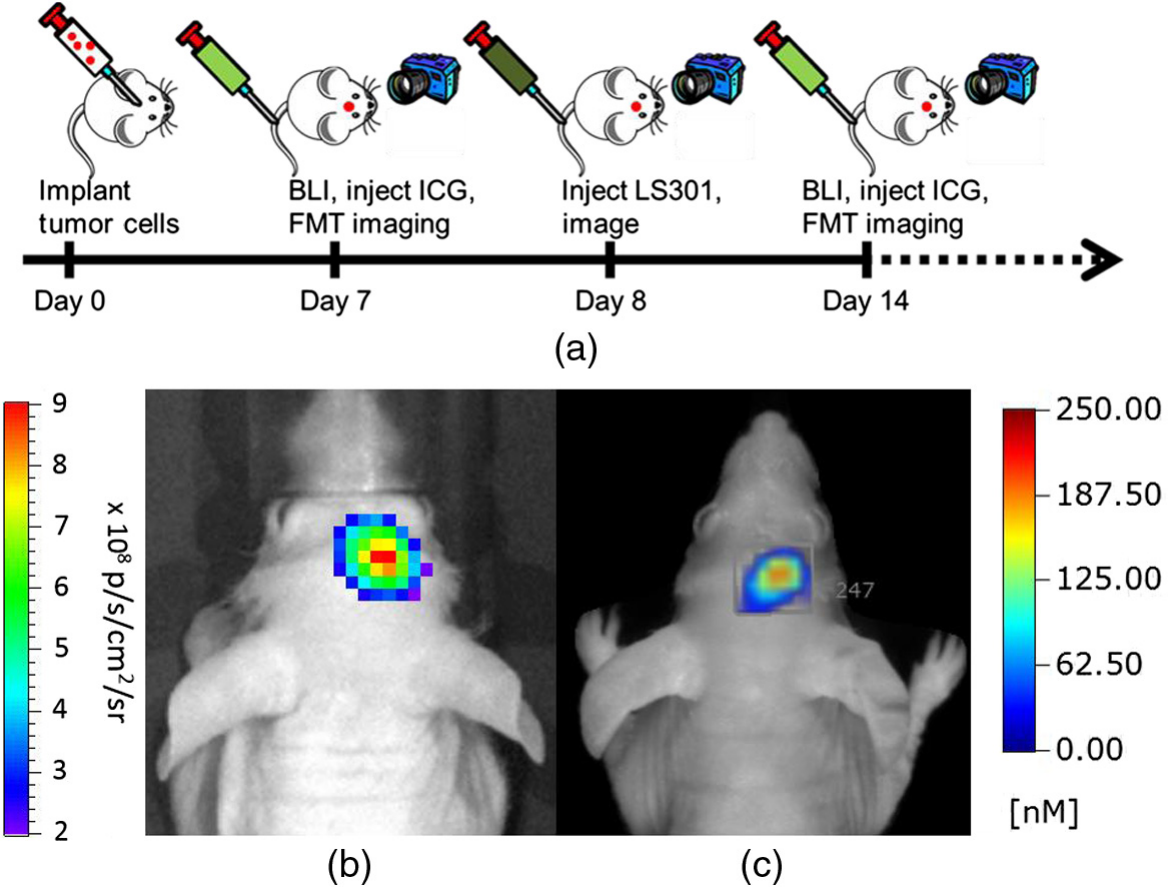
Experimental design and overview. (a) U87-GL PDE7B H217Q cells were stereotactically implanted and then mice were imaged weekly with BLI and FMT. Mice were injected with ICG and imaged several times over 24 h and then injected with LS301 and imaged for 24 h for up to 7 weeks. (b) Representative BL image with (c) corresponding FMT image

Stereotactically implanted brain cancer cells expressing a bioluminescent reporter (U87-GL-GFP PDE7B H217Q cells) allowed for facile longitudinal localization and monitoring of tumor burden using a commercialBLI system,^25^^,^^26^ and FMT was used to quantify the delivery of ICG and LS301 to tumors (Fig. 1). Despite the similarity of their spectral properties, we found that ICG completely cleared from the brain and surrounding tissues within 24 h, allowing us to repurpose the same animal for monitoring the distribution of LS301 in the brain. In addition, preinjection images were acquired before each injection, which allowed for background subtraction of any residual fluorescence. Given this experimental design, we longitudinally examined the uptake of ICG and LS301, which helped to mitigate the effects of interanimal variability.

### 3.1 Comparison of ICG and LS301 FMT Images in Tumor-Bearing and Sham-Treated Mice

**Fig. 2:**
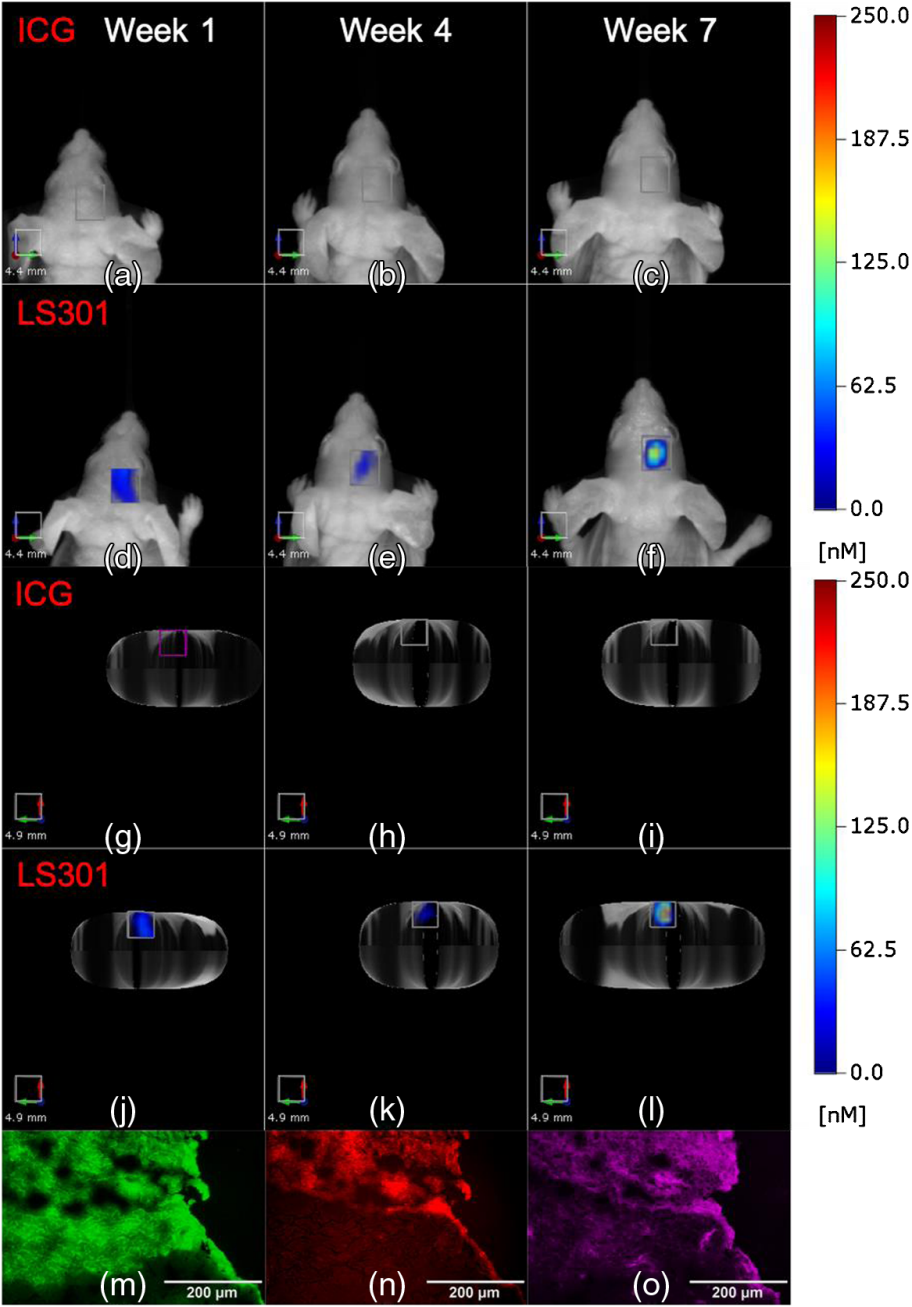
Comparison of (a)–(c), (g)–(i) ICG and (d)–(f), (j)–(l) LS301 uptake in tumor-bearing mice imaged at 24 h postinjection on weeks 1, 4, and 7 after tumor initiation. (a)–(f) Coronal projections of images with the corresponding axial projection images in (g)–(l) viewed head first for improved visualization. Epifluorescence microscopy of U87-GL PDE7B H217Q tumors harvested at 72 h postinjection of LS301 at 7 weeks post-tumor implantation. (m) GFP fluorescence of tumors (green). (n) NIR fluorescence of brain tissue (red). (o) p-ANXA2 stain of the tumor (magenta).

Previous reports demonstrated a high tumor-to-background fluorescence 24 h after intravenous injection in GBM models.^11^^,^^12^ Recently, we showed that LS301 also provides excellent tumor contrast 24 h postintravenous injection in mice.^14^^–^^16^ Given these findings, we compared LS301 and ICG uptake in brain tumor-bearing mice at 24 h postinjection. We validated the uptake pattern by time-course imaging of the imaging agents up to 24 h and comparing the mean tumor ROI fluorescence in tumor-bearing mice to naïve and sham-treated controls. While enhancement of LS301 uptake relative to these controls is observed at 24 h postinjection at week 7 post-tumor implantation, accumulation of ICG in tumors is not statistically significant relative to controls at any time point recorded. LS301 reveals focal fluorescence in tumor tissue 24 h after LS301 injection at weeks 1, 4, and 7 (Fig. 2). Epifluorescence microscopy of brain tumor sections harvested at 72 h postinjection of LS301 at 7 weeks postimplantation shows the presence of GFP signal [Fig. 2(m)] and NIR fluorescence from LS301 [Fig. 2(n)] from the tumor in the cerebral cortex. Subsequently, immunohistochemistry of p-ANXA2 expression demonstrates excellent agreement between areas of LS301 accumulation and p-ANXA2 expression in the tumor [Fig. 2(o)].

**Fig. 3:**
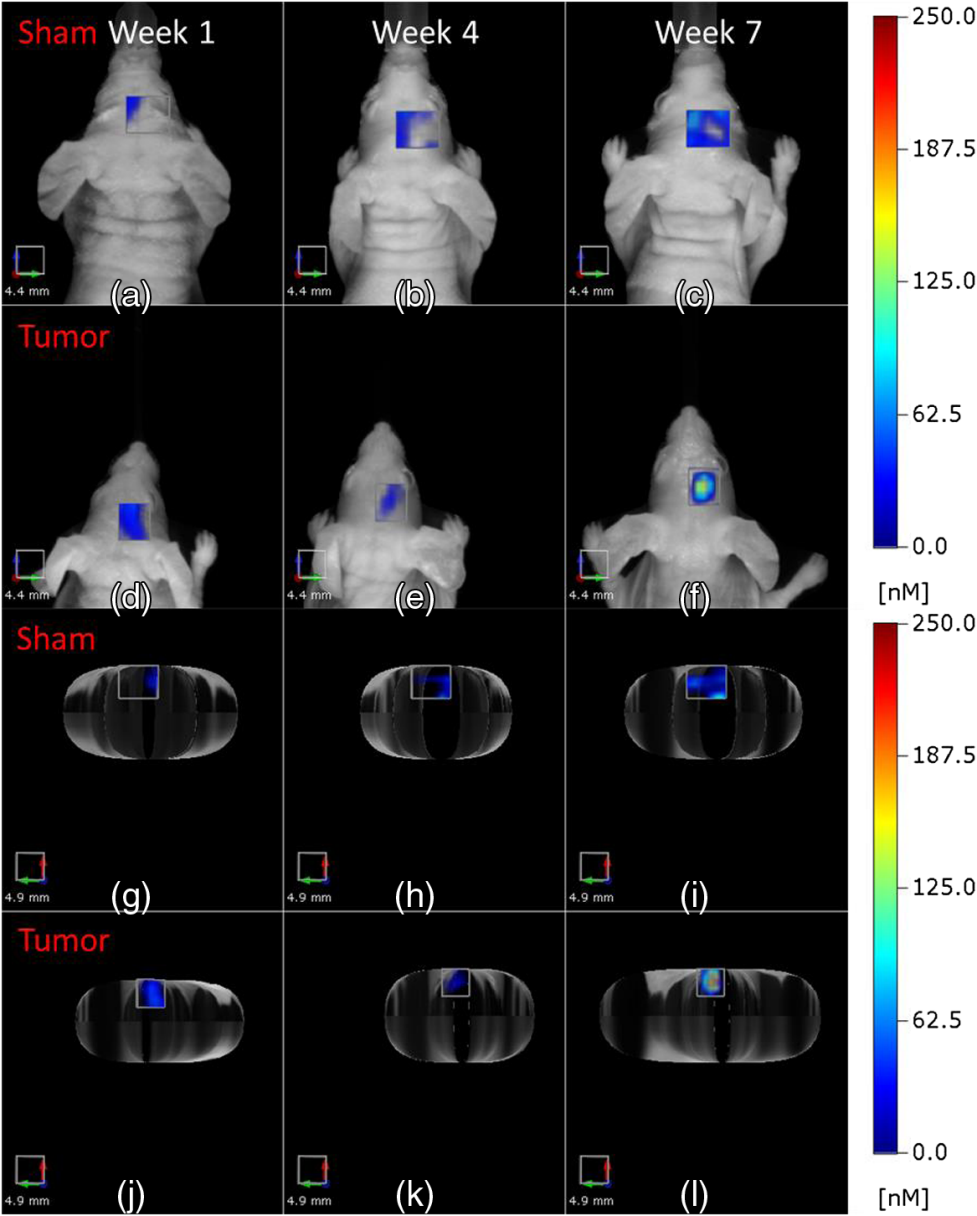
Comparison of FMT images of tumor ROIs in tumor-bearing and sham-treated mice. Representative images of (a)–(c), (g)–(i)  sham-treated and (d)–(f), (j)–(l) tumor-bearing mice imaged at 24 h post-LS301 injection on weeks 1, 4, and 7 after tumor initiation or sham injection. (a)–(f) Coronal projections of images with the corresponding axial projection images in (g)–(l).

Further analysis of the data suggests that LS301 fluorescence may not completely co-localize with the tumor tissue. To account for nonspecific fluorescence in the tumor ROI, naïve and sham-treated controls were injected with ICG and LS301. Following the experimental workflow shown in Fig. 1, naïve mice received stereotaxic cortical injections of PBS and were imaged weekly up to 7 weeks to match data from the tumor cohort. Our result shows a clear difference in the distribution of LS301 at 24 h postinjection compared to sham-treated mice (Fig. 3). The sham-treated mice have low and diffuse LS301 fluorescence in the ROI similar to tumor implantation site, whereas tumor-bearing mice display a focal fluorescence distribution. This is best visualized in the axial view [Figs. 3(j)–3(l)]. However, the mean fluorescence in the ROI for both tumor and sham was not statistically distinguishable in the early time points until 7 weeks postimplantation, reflecting the poor penetration of LS301 into the brain until tumor degrades the BBB.

### 3.2 Quantification of Bioluminescence and Tumor ROI Fluorescence

**Fig. 4:**
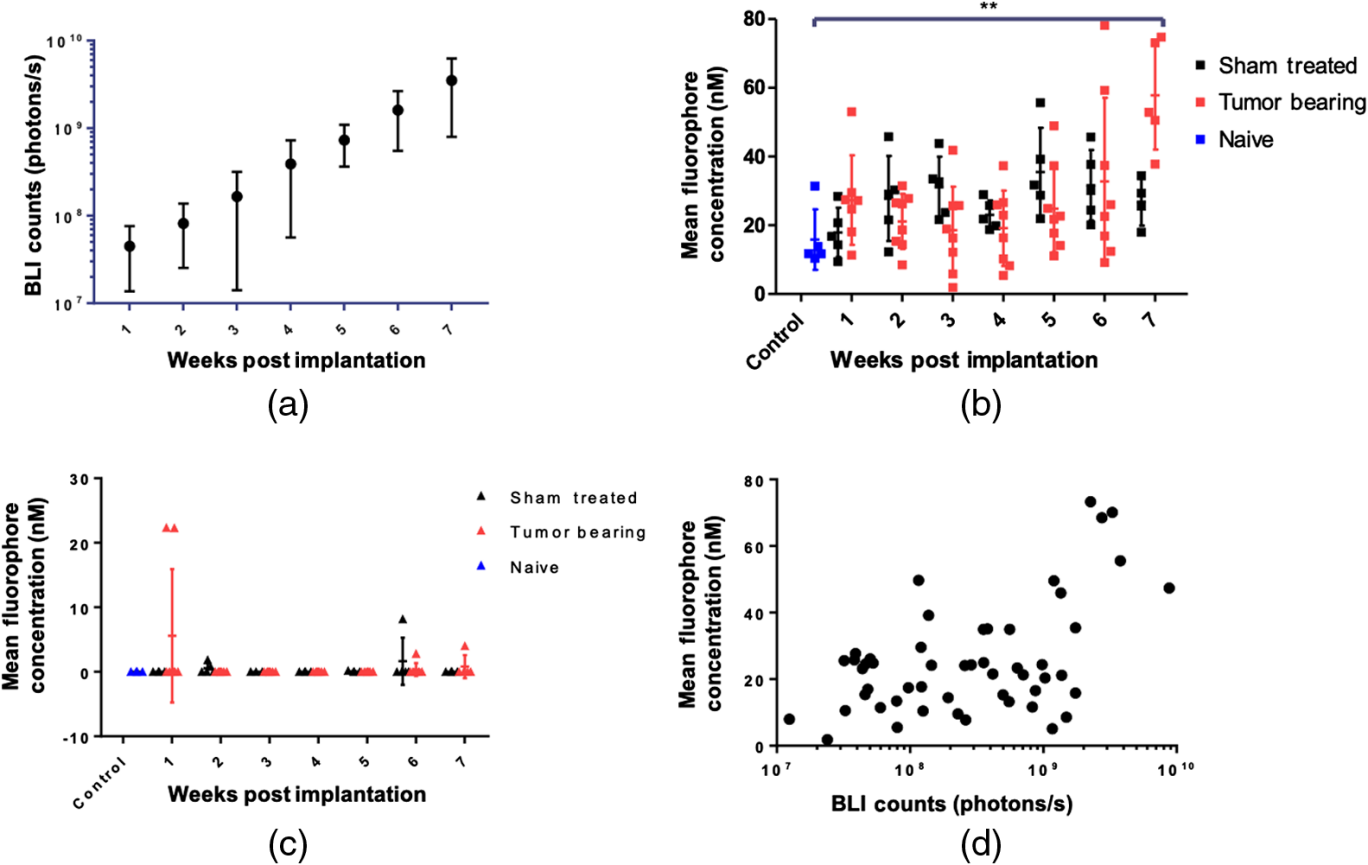
Quantification of tumor fluorescence and bioluminescence. (a) Longitudinal quantification of tumor bioluminescence. (b) Longitudinal quantification of LS301 mean fluorescence at 24 h postinjection in naïve, sham-treated, and tumor-bearing mice. (c) Longitudinal quantification of ICG mean fluorescence at 24 h postinjection in naïve (n=5), sham-treated (n=5), and tumor-bearing mice (n=8). (d) Longitudinal quantification of LS301 mean fluorescence as a function of BLI counts. BLI data are shown on log scale for ease of viewing.

Longitudinal bioluminescence quantification demonstrates a log-linear growth of the tumors [Fig. 4(a)] but LS301 fluorescence at 24 h postinjection remains stable from weeks 1 to 6 and then begins to separate from the sham control at week 7 [Fig. 4(b)]. A statistically significant difference in LS301 uptake is only achieved at 7 weeks postimplantation between the naïve and tumor-bearing cohort. In addition, a clear difference between LS301 fluorescence in the sham-treated and tumor-bearing cohorts is only apparent at week 7, although the diffuse nature of background LS301 fluorescence across different animals prevented the attainment of statistical significance after correcting for multiple comparisons (p=0.058). Comparing LS301 fluorescence at 24 h postinjection as a function of bioluminescence signal shows a weak linear correlation [$r^{2}=0.2797$, p<0.0001, Fig. 4(d)], which could be attributed to a combination of factors that include LS301 penetrance in tumor, limited access to tumor tissue in the brain, and differences in the imaging strategies. In all cases, the mean ROI fluorescence for ICG at 24 h postinjection was close to zero, with no significant difference between sham-treated and tumor-bearing mice [Fig. 4(c)].

### 3.3 Focused Ultrasound Delivery of LS301 and ICG to Brain Tumors

**Fig. 5:**
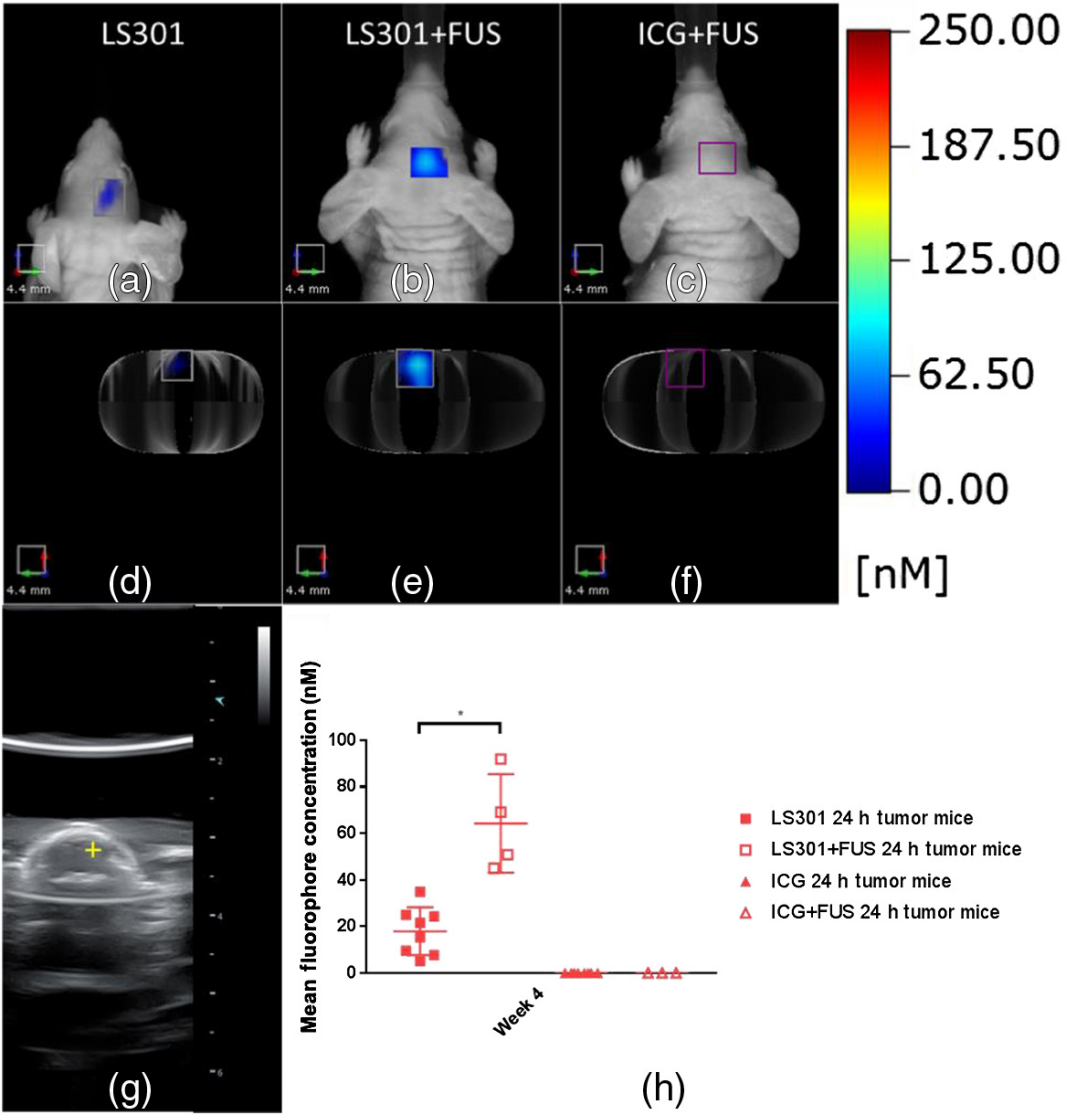
FUS delivery of LS301 and ICG at 4 weeks post-tumor initiation. (a), (d) Coronal and axial FMT projections of LS301 fluorescence at 24-h postinjection. (b), (e) Coronal and axial FMT projections of LS301 fluorescence at 24 h postinjection and FUS sonication. (c), (f) Coronal and axial FMT projections of ICG fluorescence at 24 h postinjection and FUS sonication. (g) B-mode ultrasound image showing focus of ultrasound treatment. (h) Quantification of tumor ROI fluorescence with and without FUS treatment.

The difficulty in delivering contrast agents across an intact BBB contributes to the inability to implement imaging protocols for the early detection of brain cancer. FUS with microbubbles has been shown to transiently disrupt the BBB.^27^ To explore this phenomenon, we injected LS301 into mice 4 weeks after tumor initiation when the BBB is intact, followed by FUS treatment and imaging 24 h post-LS301 injection. Four days later, those same mice were again treated with FUS, injected with ICG, and imaged 24 h later. Figure 5(g) shows the focus of the FUS treatment, which was based on BLI imaging and the stereotaxic coordinates of the tumor injection. Quantification of the mean tumor ROI fluorescence for mice injected with LS301 or ICG is shown in Fig. 5(h). Significance testing between groups with and without FUS treatment was carried out using a Student’s t-test with Welch’s correction.^28^ We found that FUS enhances the uptake of LS301 compared with mice the non-FUS-treated mice [Figs. 5(a)–5(f)]. Even with FUS, ICG still clears rapidly from the tumor region and was not visible by 24 h, confirming that tumor-targeting molecular probes confer higher uptake and retention in brain tumors if BBB permeation is feasible.

### 3.4 Comparison of LS301 and Alexa Fluor 680 Transferrin

**Fig. 6:**
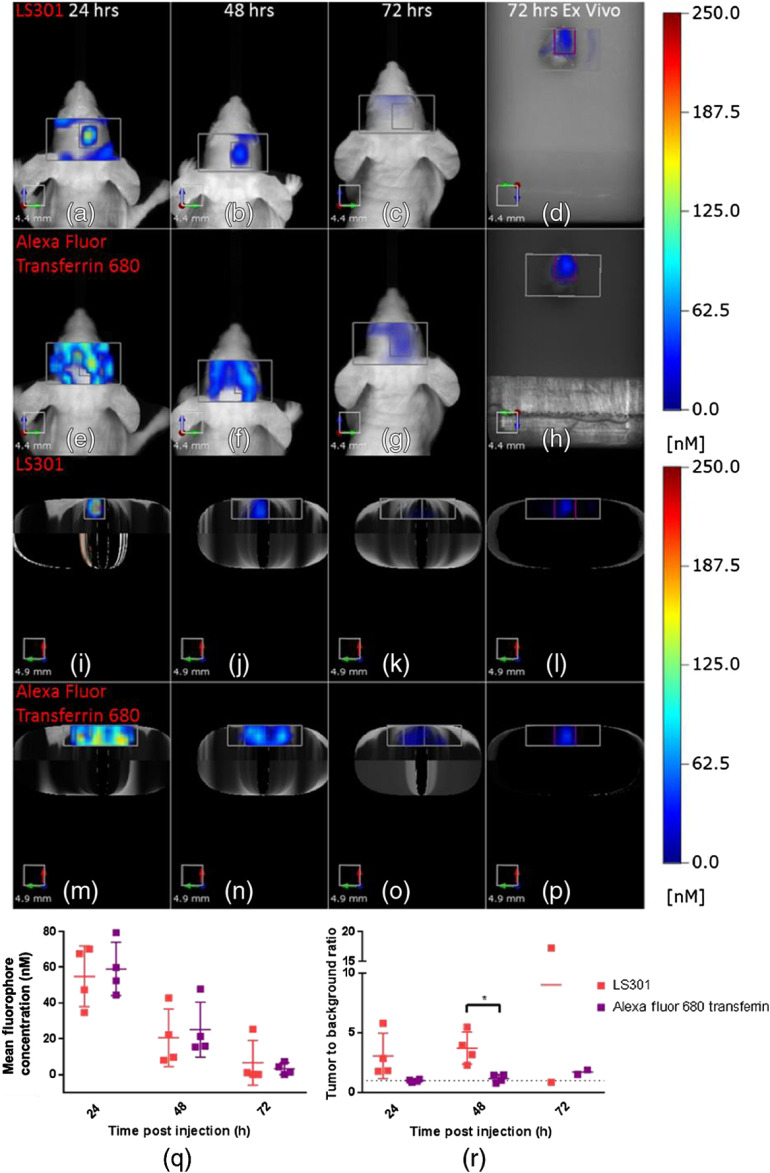
*In vivo* and *ex vivo* images of LS301 (6-nmol injection) and Alexa Fluor 680 transferrin (2 nmol injection). (a)–(d) Coronal projection of LS301 fluorescence in the tumor ROI and surrounding area. (e)–(h) Coronal projection of Alexa Fluor 680 transferrin fluorescence in the tumor ROI and surrounding area. (i)–(l) Axial projection of LS301 fluorescence in the tumor ROI and surrounding area. (m)–(p) Coronal projection of Alexa Fluor 680 transferrin fluorescence in the tumor ROI and surrounding area. (q) Quantification of tumor ROI mean fluorescence. (r) Tumor-to-background ratio.

A previous report showed that a transferrin-imaging agent is able to target gliomas selectively.^21^ In this study, we compared the distribution of the reported glioma-targeting agent, AF-Tf and LS301. The differences in their imaging spectral windows (670/690 to 740 nm for AF-Tf and 780/>805  nm) allowed us to differentiate the spectral signatures of both agents with the dual excitation and emission features of the FMT system. To ensure that the imaging agents had access to the tumors, we coinjected a solution of LS301 (6 nmol) and AF-Tf (2 nmol) in PBS in a subset of the tumor-bearing mice to compensate for differences in the fluorescence quantum yields of Alexa Fluor (0.36) and cypate dye (∼0.1) used to prepare AF-Tf and LS301, respectively.^19^^,^^29^ We imaged for up to 72 h at 7 weeks post-tumor implantation, when the BBB is compromised. Fluorescence was observed in the tumor ROIs as well as the surrounding areas that encompass the top 3 to 4 mm of the brain, which we used as background [Figs. 6(a)–6(p)]. Time course imaging and comparison of LS301 with AF-Tf show strong signals in the tumor ROI for the molecular probes. The faster clearance of LS301 from nontumor tissue compared to AF-Tf enhances the tumor-to-background contrast. Quantitative analysis of the data shows that the high background AF-Tf fluorescence made it difficult to distinguish the tumor from surrounding tissue at the 24- and 48-h time points [Fig. 6(r)]. This yields an apparent difference in the tumor-to-background ratio between LS301 and AF-Tf at 24 h, with a statistically significant difference at 48 h postinjection (p<0.05), which is in agreement with the *ex vivo* results [Figs. 6(d), 6(h), 6(l), and 6(p)]. AF-Tf appears to highlight the brain vasculature given the tortuous shape of the signal. This pattern of AF-Tf distribution can be attributed to the high expression of transferrin receptors in the brain capillary endothelium.^30^

## 4 Discussion

Longitudinal imaging is a vital means of understanding disease pathogenesis and progression. Of all the current medical imaging systems, fluorescence imaging method is particularly suited for longitudinal imaging given its high throughput, lack of ionizing radiation, and low cost. Furthermore, the ability to monitor multiple fluorescent probes using distinct spectral bands allows for the surveillance of diverse disease processes in concert. For imaging of brain disease in small animals, FMT enables quantitative and noninvasive imaging of whole heads with depth resolution, which is not readily attainable by planar reflectance fluorescence or BLI.

In this work, we devised a workflow that allowed us to compare the uptake of passive (ICG) and active (LS301) tumor-targeted imaging agents. We find that the tumor-avid molecular probe, LS301, selectively accumulates in the brain tumor region for a prolonged period, compared to the perfusion-based uptake and rapid clearance from the entire brain. This finding is contrary to reports where ICG fluorescence was shown to retain in brain tumors up to 24 h postinjection and the second NIR window of ICG was employed to provide fluorescence guidance for glioma resection. ^11^^,^^12^ A variety of reasons could explain this discrepancy. Possibly, the fluorescence camera and setup used in the previous studies may be more sensitive in the NIR window than the FMT system we used. Another major consideration is the animal models used. Our glioma model was designed to provide bioluminescence signal for noninvasive BLI and the cell transformation may alter the pathophysiological properties of the glioma tumor cells. We used the lowest sensitivity setting of the FMT in this study to avoid oversaturation of images at early time points, leading to a significant loss of detection sensitivity at later imaging time points with the same settings. Future studies will explore the use of dynamic thresholding to determine if increasing the sensitivity enables detection of low concentration of fluorescent imaging agents at later time points. Unlike previous studies that used ICG dose of 5  mg/kg, we employed a considerably smaller dose (∼0.233  mg/kg) in this study. At equivalent concentration, this study demonstrated that LS301 fluorescence was still detectable in tumors, demonstrating the highly tumor-selective features of the molecular probe and suitability for NIR fluorescence-guided glioma resection.

Longitudinal imaging demonstrated that a statistically significant difference in LS301 uptake is achieved at 7 weeks postimplantation between the naïve and tumor-bearing cohort when tumor-induced BBB degradation occurred. In addition, an apparent difference between LS301 fluorescence in the sham-treated and tumor-bearing cohorts is only observed after 7 weeks as well. This is in contrast to other reports where tumors can be visualized by intravenous injection of NIR fluorescent dyes as early as 1 week after tumor initiation.^31^^,^^32^ These previous studies injected about a million cells in 5  μL compared with 50,000 cells in 2  μL used in this study. Therefore, it is reasonable to conclude that the higher initial number of cells can delay the reestablishment of BBB integrity, accounting for the different outcomes. Further, our approach had minimal perturbation of the BBB, as demonstrated by the rapid reestablishment of the BBB following tumor implantation. The focal nature of FUS permeabilization of the BBB probably does not perturb the tumor tissue, thereby minimizing the nonspecific accumulation of imaging agents in tumor and brain tissues. The published studies also did not include sham controls, making it difficult to determine how the implantation procedure alone impacts the uptake of molecular probes in tumors.

Although there was no significant difference between tumor-bearing and sham mice injected with ICG, we did observe that two animals had much higher uptake than other mice in any group at 1-week post-tumor initiation [Fig. 4(c)]. This finding suggests that nonspecific uptake of passive targeted dyes may occur in some mice up to 1-week post-tumor initiation, probably caused by the rapid proliferation of the tumor cells that further delays the reestablishment of BBB and disrupts organized vascular structure. This finding is also consistent with a previous report studying the uptake of nonspecific fluorescent molecules in a rat model of glioma, 1 week after tumor initiation, using fluorescence microscopy.^33^ However, twice as many cells in PBS were injected at the time of implantation compared with what was used in this study. This may account for the discrepancy between the results presented here and those reported previously. Variability of results is an expected outcome in most longitudinal cancer imaging studies, which depends on multiple factors, including the age of animals, tumor size, contrast agent pharmacokinetics, dosing, and instrument used. Nevertheless, the fluorescence pattern of LS301 in the tumor ROIs is clearly different between sham and tumor-bearing mice. Application of a threshold based on naïve controls or shape analysis of tumor ROI fluorescence may help to further distinguish tumor from background.^34^

We also demonstrated that FUS-assisted delivery of LS301 can significantly enhance tumor contrast at time points in tumor progression when BBB is intact, preventing access of intravenously administered molecular probes to brain tumors. This finding illustrates how a combination of tumor-targeting molecules and transient BBB disruption can additively augment both tumor uptake and retention in the brain. The transient nature of FUS-mediated BBB disruption provides a way to deliver various molecular probes to orthotopic brain tumors without the need to develop new chemical formulations to pass the BBB. Furthermore, delivery of fluorescent probes to brain tissue may also enable molecular imaging of other diseases of the central nervous system, which do not exhibit compromised BBB integrity.

Lastly, we sought to compare LS301 with another tumor-targeted molecular probe, AF-Tf. These results show faster clearance of LS301 from background tissues and thus it achieves higher tumor-to-background ratios than AF-Tf throughout the imaging time points examined. A combination of large size and the abundance of transferrin receptors expression on brain capillaries could account for the long circulation time and slow clearance of AF-Tf from nontumor tissue.^30^ This work also demonstrated the multiplexing capability of fluorescence imaging, which has not been utilized in previous studies employing FMT to image GBM.^10^^,^^31^^,^^32^^,^^35^ Future studies could expand the use of this strategy to include glioma models-expressing iRFPs^36^ in concert with exogenous contrast agents to validate tumor positioning and monitor multiple brain tumor biomarkers such as integrin expression^37^ or protease activity^31^^,^^32^ as a function of tumor progression or response to treatment.

## 5 Conclusion

We implemented FMT to longitudinally monitor the delivery of passive and active targeted fluorescent molecular probes in an orthotopic brain tumor model. We observed that FUS enhances the delivery and retention of an actively targeted (LS301) but not a nontumor selective (ICG) molecular probe. Finally, we demonstrated LS301 has longer retention in tumors and faster clearance from background tissue with respects to ICG and AF-Tf, respectively. Our results lay the foundation for using FMT in combination with FUS to determine the effects of tumor progression on BBB, interrogate the selectivity of different imaging agents for brain cancer, and detect brain lesions at early stages of pathogenesis.
